# Cerebral Palsy—Trends in Epidemiology and Recent Development in Prenatal Mechanisms of Disease, Treatment, and Prevention

**DOI:** 10.3389/fped.2017.00021

**Published:** 2017-02-13

**Authors:** Moshe Stavsky, Omer Mor, Salvatore Andrea Mastrolia, Shirley Greenbaum, Nandor Gabor Than, Offer Erez

**Affiliations:** ^1^Faculty of Health Sciences, School of Medicine, Ben Gurion University of the Negev, Beer Sheva, Israel; ^2^Department of Obstetrics and Gynecology, University of Bari Aldo Moro, Bari, Italy; ^3^Faculty of Health Sciences, Department of Obstetrics and Gynecology, Soroka University Medical Center, School of Medicine, Ben Gurion University of the Negev, Beer Sheva, Israel; ^4^Systems Biology of Reproduction Lendulet Group, Institute of Enzymology, Research Centre for Natural Sciences, Hungarian Academy of Sciences Budapest, Budapest, Hungary; ^5^Maternity Private Department, Kutvolgyi Clinical Block, Semmelweis University, Budapest, Hungary; ^6^First Department of Pathology and Experimental Cancer Research, Semmelweis University, Budapest, Hungary; ^7^Faculty of Health Sciences, Maternity Department “D”, Division of Obstetrics and Gynecology, Soroka University Medical Center, School of Medicine, Ben Gurion University of the Negev, Beer Sheva, Israel

**Keywords:** cerebral palsy, neuroinflammation, progesterone, *N*-acetyl cysteine, nanoparticles, magnesium sulfate, birth asphyxia, intrauterine infection

## Abstract

Cerebral palsy (CP) is the most common motor disability in childhood. This syndrome is the manifestation of intrauterine pathologies, intrapartum complications, and the postnatal sequel, especially among preterm neonates. A double hit model theory is proposed suggesting that an intrauterine condition along with intrapartum or postnatal insult lead to the development of CP. Recent reports demonstrated that treatment during the process of preterm birth such as magnesium sulfate and postnatal modalities such as cooling may prevent or reduce the prevalence of this syndrome. Moreover, animal models demonstrated that postnatal treatment with anti-inflammatory drugs coupled with nanoparticles may affect the course of the disease in pups with neuroinflammation. This review will describe the changes in the epidemiology of this disease, the underlying prenatal mechanisms, and possible treatments that may reduce the prevalence of CP and alter the course of the disease.

## Introduction

Cerebral palsy (CP) is the most common motor disability in childhood (1). CP is a heterogeneous group of clinical syndromes that describe permanent disorders of movement and posture. It is characterized by abnormal muscle tone, posture, and movement, thereby limiting the activity of the affected person. The motor disorders of CP are often accompanied by disturbances of sensation, perception, cognition, communication and behavior, epilepsy, and secondary musculoskeletal problems (2). These disorders are attributed to non-progressive disturbances in the developing fetal brain (2), alteration in fetal development (2), pathologic intrauterine processes (3–8), or considered as prematurity complication (9). Although CP itself is not a progressive disease, its clinical expression may change over time as the brain matures. In this review, we describe the changes in the epidemiology of this disease and the underlying prenatal mechanisms as well as possible treatments that may reduce its prevalence and alter the course of the disease.

## Epidemiology of CP

Population-based studies from around the world report that the prevalence estimates of CP range from 1.5 to more than 4 per 1,000 live births or children of a defined age range (10–14). The overall birth prevalence of CP is approximately 2 per 1,000 live births (15–17).

A population-based study from the USA reported a relatively stable rate of spastic CP, 1.86/1,000 in 1985 to 1.76/1,000 in 2002. Of interest, there were racial disparities in the changes of the prevalence of CP along that time period: while in non-Hispanic White population, the overall prevalence declined from 1.65/1,000 in 1985 to 1.34/1,000 in 2002, the prevalence of CP in non-Hispanic Blacks increased from 2.29/1,000 in 1985 to 2.34/1,000 in 2002 (18). The 2012–2013 National Survey of Children’s Health (NSCH) and the 2011–2013 National Health Interview Survey (NHIS) determined the prevalence of CP through parents’ reports among children aged 2–17 years. These surveys found a CP prevalence per 1,000 live births that ranged from 2.6 in the NSCH to 2.9 in the NHIS (19). In a population-based study from Iceland (20), the prevalence of CP per 1,000 live births did not change significantly from 1990 to 2003, which stayed between 2.2 and 2.3. However, it decreased from 1.5 to 0.9/1,000 live births for children born at term, was stable for preterm births, and increased from 33.7 to 114.6/1,000 live births for very preterm births. An observation that may explain some of these findings is the parallel increase in the rate of cesarean sections. The Australian Cerebral Palsy Register, including information from 1993 to 2006 reported an overall CP prevalence of 2.1 per 1,000 live births with high prevalence in multiples (7 per 1,000 live births) and in extremely low birthweight neonates (e.g., for birthweight <1,000 g, the prevalence was 50 per 1,000 live births) (21). Overall, the total rate of CP is relatively stable, yet the contribution of prematurity and its complication to the prevalence of this syndrome are steadily increasing due to improvements in obstetric and neonatal care.

## Risk Factors for CP

Cerebral palsy can be derived from any event that will affect the fetal and neonatal developing brain. Indeed, congenital malformations, fetal growth restriction, multiple gestations, infection during the fetal and neonatal period, birth asphyxia, preterm delivery, untreated maternal hypothyroidism, perinatal stroke, and thrombophilia were all recognized as risk factors for CP (22–25). Premature birth, especially before 28 weeks of gestation, is the leading risk factor for the development of CP (26). The birth prevalence of CP is far higher in preterm than in term infants, increases with decreasing gestational age at delivery (27, 28), and can reach up to 15% among preterm neonates who were born between 24 and 27 weeks of gestation (29). Indeed, the prevalence in 1,000 live births of CP among neonates who were born prior to 28 weeks of gestation is 82, and it decreases to 1.4 at 36 weeks of gestation (30). Of interest, although preterm delivery is a well-established risk factor for CP, a recent study suggests that postterm pregnancy at 42 weeks or later is also associated with an increased risk of this condition (31).

### Placenta-Mediated Pregnancy Complications

The impact of fetal growth restriction on the prevalence of CP is well established. The prevalence of this disease among neonates weighing <1,500 g is 59.2/1,000 live births, in comparison to 1.33/1,000 live births among those weighing >2,500 g (30). This association is independent of gestational age at delivery and extenuated by the presence of congenital anomalies, especially of the central nervous system (32). In an Australian population-based study (33) of singleton pregnancies delivered ≥35 weeks of gestation, the authors identified 494 children with CP. Growth restriction was prevalent in 16.5% of the neonates with CP, and the odds of these children to be growth restricted in comparison to those without was 3.5 (95% CI 2.2–5.5). A population-based study in Denmark reported a CP prevalence of 1.95/1,000 live births. CP was associated with an increased head/abdominal circumference ratio and cephalic index regardless of gestational age (34). Thus, the intrauterine processes associated with fetal growth restriction also lead to neuronal damage and subsequent CP.

The association between preeclampsia and subsequent development of CP was under constant debate. However, a recent population-based cohort study demonstrated that early-onset preeclampsia is an independent risk factor for CP (OR 8.639, 95% CI 4.269–17.480) after adjustment for fetal growth restriction and gestational age at delivery. This was not the case regarding preeclampsia at term (6).

### Congenital Malformations

The association between congenital malformations and subsequent CP is well documented. Major birth defects were the most frequently occurring risk factor in children with CP, and when combined with fetal growth restriction, they were associated with the highest relative risk (33). Congenital microcephaly is the most common birth defect in CP (35). Other non-cerebral malformations were also frequently coexisting with CP, especially cardiac (12%), urinary (5.4%), and musculoskeletal (5.4%) (36).

### Multiple Gestations

Twins have a higher frequency of malformations and CP than singletons (37, 38). Indeed, among twin gestations, an affected twin with CP increases the risk by a factor of 15 that the other one will have this syndrome as well (35). Additionally, the *in utero* death of one twin, even if it occurs early in gestation, leaves the surviving twin at markedly increased risk for CP (39). The prevalence of CP in an Australian study (40) was 1.6, 7.3, and 28 per 1,000 births in singletons, twins, and triplets, respectively. The authors also reported that in the case of co-twin death, the risk for CP increases by a factor of 8, from 12/1,000 to 96/1,000. The explanation for this observation was that the dissolving twin releases thromboplastin and emboli that may lead to brain injury of the surviving twin and subsequent development of CP.

### Perinatal Stroke

Fetal and neonatal cerebral accidents are associated with increased risk for subsequent development of CP. Indeed, among 100 full-term neonates with the diagnosis of neonatal arterial ischemic stroke, born in Switzerland between 2000 and 2010, 39% were diagnosed as having CP at the age of two (41). In a report from California, USA, of 36 children with arterial ischemic stroke, 58% had subsequent CP. The mechanisms leading to perinatal strokes are not clear; in some cases, preeclampsia was proposed as a risk factor; in addition, there are those who relate that to placental vascular disorder. The association between CP and thrombophilia is not that clear. In an Australian population-based case–control study (42), among term neonates, there was no association between a single thrombophilic mutation and CP. Among preterm neonates with the MTHFR C677T mutation, those who were homozygous and were born between 32 and 36 weeks of gestation had an odd ratio (OR) of 2.55 (95% CI 1.12–5.74), while the heterozygous ones had an OR of 1.91 (95% CI 1.01–3.66) to develop any type of CP (42). This MTHFR mutation at the homozygous state, among preterm neonates born <32 weeks of gestation, was also associated with increased risk for diplegia (OR 2.76, 95% CI 1.21–6.12), while the heterozygous was associated with a mildly increased risk for diplegia throughout gestation (OR 1.58, 95%, CI 1.02–2.45). A different mutation, MTHFR A1298C at the heterozygote state, was associated with risk reduction for diplegia in neonates born at 32–36 weeks of gestation (OR 0.16, 95% CI 0.02–0.70). Among early preterm neonates (<32 weeks of gestation), FVL homozygosity may be associated with an increase in the risk of developing quadriplegia (OR 9.12, 95% CI 0.86–53.71). Heterozygous PGM and homozygous MTHFR C677T combined were associated with quadriplegia at all gestational ages (OR 5.33, 95% CI 1.06–23.25) (42). On the other hand, an Israeli retrospective case–control study found no increased prevalence of thrombophilic mutations among children with non-stroke CP (43). The association between perinatal stroke and subsequent CP may suggest that these conditions may represent that in some of the cases, the course of progression of a disease, starting in the uterus, has a neonatal presentation as acute ischemic stroke and subsequently develop into CP. This hypothesis requires further investigation.

## Genetics Contributions to CP

There are indications of genetic involvement in CP, and its contribution is estimated to be as high as 48% of term and 24% of preterm idiopathic cases (44). Genetic investigations of CP are performed using population-genetic and pedigree inquiries. Population-based genetic studies have identified several candidate genes, whose variants were more frequent in CP patients in certain populations, some of which are associated with processes of inflammation, coagulation, and blood flow (45–47). Pedigree studies are mostly aimed at specific forms of CP that are found to be frequent in some families. Specific CP forms, such as ataxic CP, symmetrical spastic CP, and tetraplegic CP with MR, comprise the minority of CP cases but exhibit strong inheritance patterns, most often autosomal recessive (48–50). One interesting case, involving a family with repeated congenital CP, has led to the identification of a candidate gene with an imprinting-like inheritance pattern, whereas the mutation is expressed only when it is inherited from the father (51). This gene is crucial for normal fetal neurodevelopment, but its levels may also be decreased in the setting of hypoxia and low birth weight. It is suggested that this mechanism, as deduced from a pedigree data, may also play a role in sporadic CP cases (51).

## Mechanisms Leading to CP

**Figure 1:**
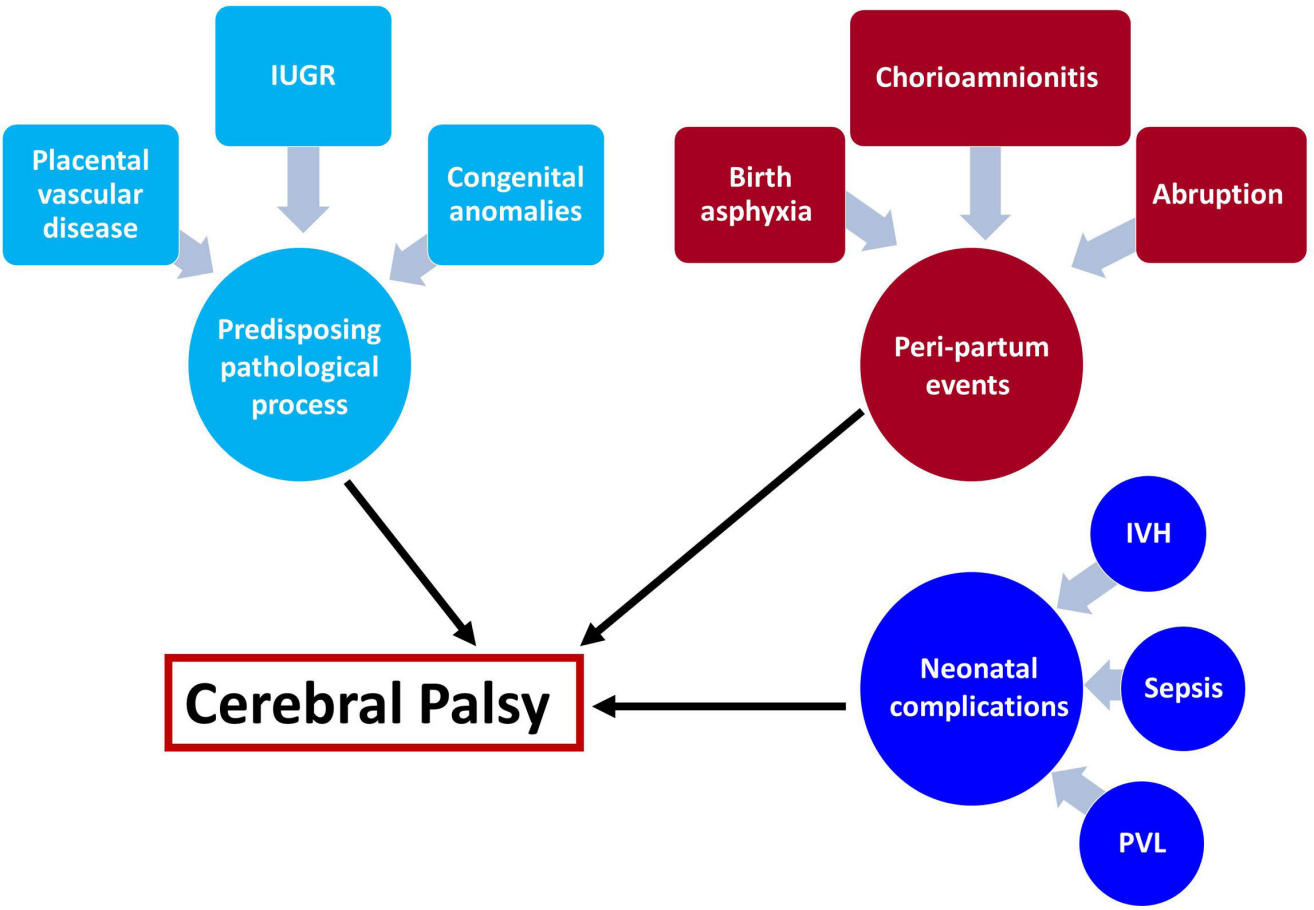
**Underlying mechanisms leading to cerebral palsy (CP)**. The mechanisms leading to cerebral palsy can be attributed to the following exposures: (1) intrauterine exposure including infection elicit a fetal inflammatory response syndrome and white matter damage in the fetus, fetal growth restriction, placental vascular disorders that are associated with vascular injuries in the fetal brain and congenital anomalies. (2) Intrapartum events that affect the fetus during the process of labor and delivery such as chorioamnionitis, birth asphyxia, and placental abruption are all acute events that are associated with further implication on the fetal/neonatal brain. (3) Post-partum exposure to infection of prematurity complications that affect the neonate and subsequently lead to CP. These events can be isolated but also combined, and the two-hit theory proposed that a neonate who suffered from hostile intrauterine environment such as infection may be further affected by acute intrapartum event such as abruption or post-partum complication and develop CP.

Understanding the mechanisms leading to a disease is a crucial part in the development of treatment and prevention modalities. We can divide the events leading to the development of CP into several categories: (1) predisposing intrauterine factors—including fetal growth restriction, placental vascular disorders, intrauterine infection/inflammation, and congenital anomalies; (2) acute events at the peripartum period—placental abruption, chorioamnionitis, and birth asphyxia; and (3) events at the neonatal period—intraventricular hemorrhage, periventricular leukomalacia (PVL), sepsis, and neonatal stroke (Figure 1). In the next sections, we will discuss the effect of predisposing intrauterine factors, especially the role of infection and inflammation.

### Placental Lesions and Their Association with CP

The placental histologic lesions enable us to identify some of the intrauterine pathological processes that may lead or contribute to the development of CP. The systematic approach of defining placental histologic lesions according to three major groups: (1) evidence of infection/inflammation; (2) maternal under perfusion; and (3) fetal vascular thrombo-occlusive disease, contributed to further defining the association between specific placental lesions and subsequent abnormal neurocognitive development of the neonate. Indeed, lesions consistent with maternal under perfusion including syncytial knots and atherosis were associated with an abnormal neonatal amplitude-integrated electroencephalographic (aEEG) recording during the first days of life of very preterm neonates (<28 weeks) (52) and a significant risk for subsequent development of CP (53). Moreover, the fetal inflammatory response in the placenta and villous edema are associated with subsequent abnormal cognitive testing in the child (53), and umbilical cord inflammation (funisitis) is associated with an increased risk for CP (54). Collectively, placental inflammatory and vascular lesions are associated with subsequent development of CP.

## Intra-Amniotic Infection and Inflammation, Fetal Inflammatory Response Syndrome (FIRS), and Subsequent CP

Although fetal growth restriction (FGR) is a greater risk factor for CP than both birth asphyxia and fetal inflammation combined (33), intra-amniotic infection and inflammation are the only mechanisms in which there is an evidence of causality between the intrauterine process and PVL. Indeed, intrauterine administration of lipopolysaccharides (LPSs) to pregnant rabbits was associated with a significant increase in the rate of PVL in the pups of the treated vs. those of the untreated rabbits (55). Evidence from amniotic fluid cultivation suggests that in general, the intrauterine cavity is a sterile environment during the mid-trimester and the prevalence of infection and or inflammation (IAI) in mid-trimester amniocentesis is 0.4% (56). This value changes substantially in the presence of pathological processes; indeed, the rate of IAI in patients with prelabor rupture of membranes is about 30% (57), in those with preterm labor is 12% with intact membranes (57), and during term labor 18% (58). The rate of intrauterine inflammation is higher than that of intrauterine infection at any given gestational age. Indeed, between 20 and 24 weeks of gestation, the frequency of intrauterine inflammation is 70%, and that of positive amniotic fluid culture is 35%; these rates decrease between 20 and 27 weeks of gestation to 30 and 20%, respectively (59). Of note, there is a ninefold increase in CP for infants of mothers who had a fever during labor (24).

The FIRS is the most serious stage of IAI. It means that the ascending intrauterine infection or the inflammatory processes (like in FIRS type 2) affect the fetus. This syndrome is characterized by systemic activation of the fetal innate immune system similar to the adult systemic inflammatory response syndrome. FIRS was originally defined in fetuses with preterm labor and preterm PROM by an elevation of the fetal plasma interleukin-6 concentration (7). Affected fetuses had evidence of multi-organ involvement, had a higher morbidity rate after adjustment for gestational age, and were more likely to have a subsequent spontaneous preterm delivery in cases of preterm PROM (7). FIRS can lead to multi-organ involvement in the following body systems; it can cause hematopoietic abnormalities (60, 61), endocrine abnormalities as a result of “stress” (62, 63), cardiac dysfunction, especially diastolic (64), and brain injury in numerous mechanisms such as high tumor necrosis factor alpha (TNFα) concentrations in PVL infants (65). Indeed, in neonates with PVL, there is an increased immunoreactivity for TNFα in the neocortex, hippocampus, basal ganglia, and thalamus (66, 67). A live demonstration of the effect of fetal neuroinflammation on neonatal neurologic functions was recently reported. In a study on New Zealand white rabbits that had an intrauterine injection of *E. coli* LPS (55), their newborn pups suffered from neurobehavioral deficits and white matter injury (WMI). Moreover, in comparison with a group that had an intrauterine injection of sterile saline, the LPS-injected newborn pups had a greater mortality rate, were hypertonic, and demonstrated significant impairment in posture, righting reflex, locomotion, and feeding. The *in utero* LPS-exposed pups also had neuroinflammation indicated by activated microglia and hypomyelination in the periventricular regions. This phenotype resembles the one found in PVL and CP (55). Therefore, collectively, the data presented here suggest that IAI has a causal role in the pathway that subsequently leads to the development of CP. This process requires fetal involvement in the form of FIRS, which in turn leads to brain dysfunction and premature labor which are suggested to cause CP (65). Finally, there are reports suggesting that CP may be the result of a two-hit model in which the mechanisms leading to the delivery of an affected neonate (i.e., preterm birth, fetal growth restriction, and birth asphyxia) are the first hit; while neonatal complications such as sequel of prematurity, sepsis, and others are the second hit that leads to abnormal neurodevelopment and subsequent CP (68). Evidence in support of this view are the finding by Mor et al. (6) regarding the mechanisms leading to CP in neonates with preeclampsia and the findings of Korzeniewski et al. (69) that “*Chronic placental inflammation, acute fetal inflammation, and neonatal inflammation-initiating illness seem to interact in contributing risk information and/or directly damaging the developing brain of newborns delivered very preterm*.”

Some examples of this hypothesis are that SGA newborns who also had systemic inflammation were at a greater risk of a low Bayley Mental Development score than their peers who had neither SGA nor systemic inflammation (68). This shows that a first hit (SGA) contributed less to developmental delay then SGA combined with a second hit such as systemic inflammation (68). Another example is that SGA combined with early preeclampsia was more prone to cause CP then SGA alone (6).

## Encephalopathy of Prematurity (EOP)

Encephalopathy of prematurity is the leading risk factor for developing CP, indeed 20–60% of individuals who have EOP eventually develop CP (70). EOP is a complex amalgam of both destructive and developmental disturbances involving both gray matter and WMI in various brain areas resulting mainly from intrauterine hypoxia–ischemia and/or systemic infection/inflammation (71). White and gray matter lesions are the most prevalent type of injury observable in the magnetic resonance images (MRIs) of children with CP, and a quantitative measure of gray and white matter lesion burden correlates with motor and cognitive dysfunction in children with unilateral CP (72).

White matter injury is well known as PVL and is a robust prognostic factor for CP (73). PVL is associated with reactive gliosis, excitotoxicity microglial activation, the release of free radicals, and subsequent hypomyelination. This type of injury is divided into cystic and diffuse. Cystic WMI are necrotic lesions with a loss of cellular elements partially filled by glial scars that can be detected by ultrasound. Its prevalence is declining, and it is now observed in only 5% of very-low-birthweight survivors. Diffuse WMI is now the most common type of lesion (observed in 90% of infants with PVL and in 50% of very-low-birthweight survivors) and is visualized *via* MRI. Rodent prenatal ischemia models have all reported diffuse WMI in various brain areas as a finding consistent with EOP and CP (74).

Gray matter injury is related to neuronal and axonal degeneration including the somatosensory pathways. Several studies suggest that abnormal somatosensory inputs may be a causal factor of motor impairments in CP (71).

## Can We Prevent or Reduce the Rate of CP?

**Figure 2:**
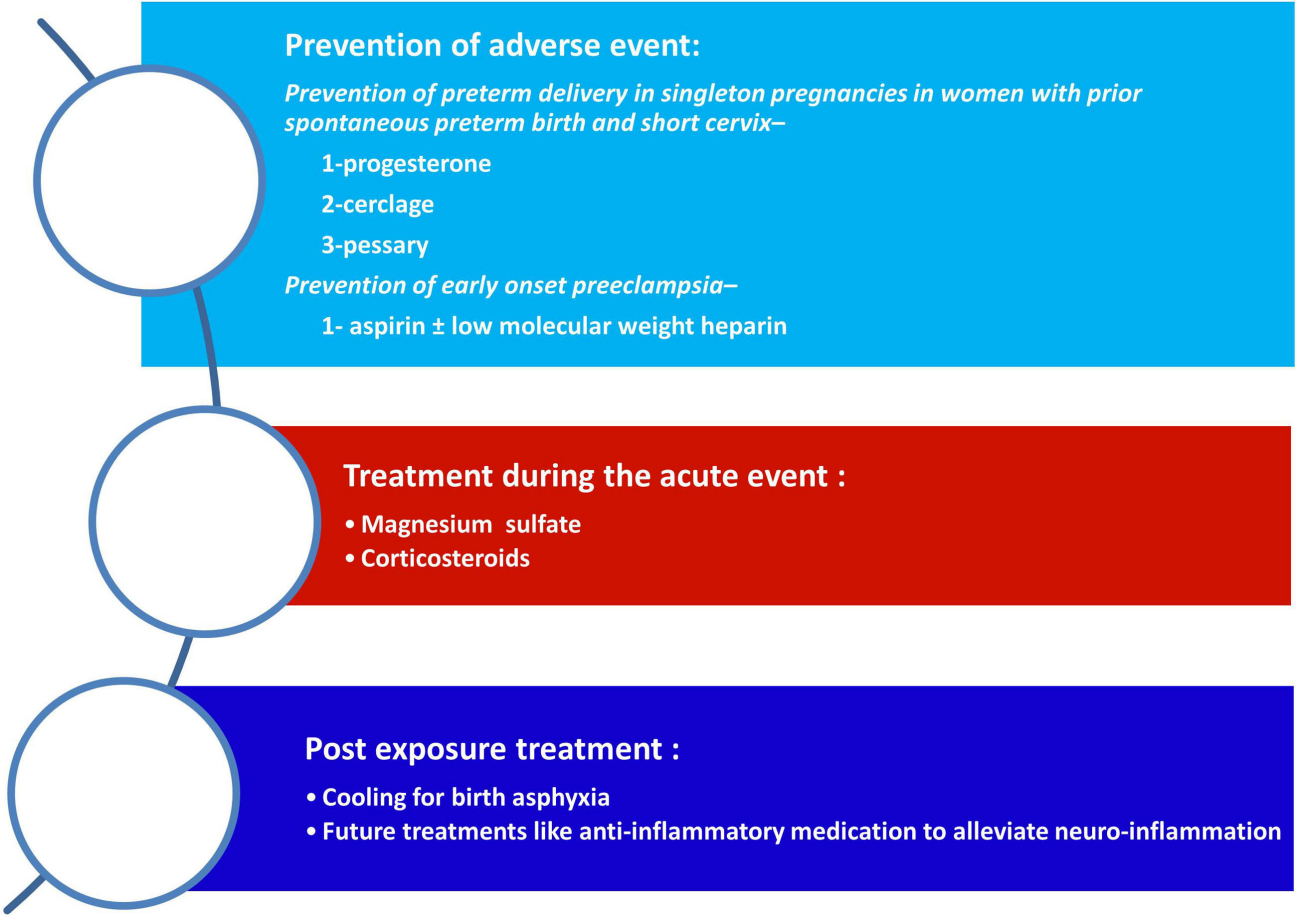
**Strategies for the reduction of cerebral palsy (CP)**. The strategies to reduce the rate of CP are as follows: (1) prevention of adverse events during pregnancy mainly of preterm birth (spontaneous or indicated); (2) the administrations of supportive medical treatment during an acute event such as magnesium sulfate of betamethasone during preterm labor; and (3) postexposure treatment to reduce the neurologic injury.

Any intervention that will lead to modification of the risk factors for CP as well as for the prevention or treatment of the underlying mechanisms that leads to this syndrome eventually will affect its prevalence. Therefore, we have three main approaches that will enable to reduce the rate of CP: (1) prevention of risk factors such as preterm birth; (2) affecting the disease process itself such as treatment with magnesium sulfate of patients who are about to deliver at early preterm; and (3) postexposure treatment of the affected neonate such as cooling of neonates with birth asphyxia (Figure 2). In the following paragraphs, we will discuss these strategies in relation to the most prominent risk factor for CP today, and that is preterm birth.

### Prevention of Risk Factors

The basis of an effective preventive strategy of any disease is represented by setting the goals which we would like to achieve. In Obstetrics, this is either to avoid the development of the disease or to alleviate its clinical presentation in a way that will minimize maternal and fetal/neonatal morbidity and mortality. A basic tool for a successful preventive program is the ability to identify patients at risk and to tailor the treatment according to the mechanisms of disease. A good example for this is the progress made during the last decade in the prevention of spontaneous premature birth. This progress is relevant to the potential reduction of CP since about half of the cases of this disease are due to prematurity and its complications. Indeed, the evidence that 17α-hydroxyprogesterone caproate can prevent recurrent preterm birth was presented at the end of the twentieth century (75, 76). Meis et al. (77) demonstrated that it can also improve neonatal outcome and turn it into the drug of choice for the secondary prevention of recurrent preterm birth. The second step in that direction was the fact that women who have a short cervix with or without a history of premature birth can benefit from vaginal progesterone for the prevention of premature birth and improvement of neonatal outcome (78–80). This finding led to the development of primary prevention of preterm birth in women with a short cervix. Overall, the administration of progesterone for the prevention of preterm birth reduced its rate in about 50% (79, 81). A cervical cerclage is an additional tool for the prevention of recurrent preterm birth in women with a short cervix. The Maternal-Fetal Medicine Units Network of the National Institutes of Health of the USA demonstrated in a multicenter randomized trial that in women with prior spontaneous preterm birth with a short cervix (<25 mm), cerclage reduced pre-viable birth and perinatal mortality but did not prevent deliveries <35 weeks, unless cervical length was shorter than 15 mm, in which recurrent preterm birth was reduced by 40–50% (82). Comparisons of the use of vaginal progesterone or cerclage were found to be equally efficacious in the prevention of preterm birth in women with a sonographic short cervix in the mid-trimester, singleton gestation, and previous preterm birth. Selection of the optimal treatment needs to consider adverse events, cost, and patient/clinician preferences (83). The use of cervical pessary for the prevention of preterm birth was also described. A randomized controlled trial, which studied pregnant women (aged 18–43 years) with a cervical length of 25 mm or less and divided them into two groups (one using cerclage and one that did not), found that spontaneous delivery before 34 weeks of gestation was significantly less frequent in the pessary group than in the expectant management group [12 (6%) vs. 51 (27%), OR 0.18, 95% CI 0.08–0.37; *p* < 0.0001]. No serious adverse effects associated with the use of a cervical pessary were reported. Therefore, cervical pessary use could prevent preterm birth in a high-risk population with a short cervical length (84). Once again, when compared, cerclage, vaginal progesterone, and pessary appear to have similar effectiveness as management strategies in women with singleton pregnancy, previous spontaneous preterm birth, and a short cervix (85).

Aside from the prevention of spontaneous preterm parturition the reduction of placental-mediated pregnancy complications that necessitate indicated preterm birth can also attribute for the reduction in CP prevalence. Indeed, our group demonstrated an association between early-onset preeclampsia as well as IUGR and subsequent development of CP (6). Therefore, any intervention that will reduce the rate of early-onset disease or the development of IUGR will have a chance to reduce the risk of CP. Preliminary evidence suggests that aspirin (86), as well as low molecular heparins (87, 88), may be effective in the secondary prevention of placental-mediated diseases.

Collectively, all these treatments may affect the overall incidence of CP by reducing the rate of preterm deliveries. However, as of today, there are neither reports in the literature to support this assumption nor any studies that were conducted to address this question. Moreover, although the rate of preterm delivery declined in the past few years, the major fraction in which the change occurred was that of the late preterm birth, although the CP is more prevalent among the early preterm birth and a change in this group may be observed only in a few more years.

### Affecting the Disease Process

Recent years yielded evidence that the treatment of women with early preterm parturition with magnesium sulfate (MgSO_4_) can have a protective effect against the subsequent development of CP (89, 90), especially the severe phenotype (90). The large randomized controlled trial conducted by the Maternal-Fetal Medicine Units Network of the NICHD included 2,241 women with threatened preterm birth who were randomized to magnesium vs. placebo. In the primary composite outcome of neonatal death and CP, there was no statistical difference between the study groups. However, a secondary analysis revealed that the rate of moderate and severe CP was reduced substantially (relative risk, 0.55; 95% CI 0.32–0.95) (90). This observation was further supported by several meta-analyses (91–93). One of the meta-analyses reported that MgSO_4_ given to women at risk of premature birth reduced the risk of CP by 30% (91). In addition, the authors reported that the number of mothers needed to treat to prevent one case of CP was 52 and that the cost to prevent one case of CP was 10,291$ (91). Therefore, MgSO_4_ should be considered for use in patients at high risk of delivery before 32 weeks of gestation (94). With this evidence at hand, a secondary analysis of the study by Kamyar et al. reported that in neonates with clinical chorioamnionitis, the beneficial effect of MgSO_4_ could not be demonstrated (95). Thus, in the general parturient of population who are at risk for preterm birth, the current approach is the antenatal administration of MgSO_4_ is associated with a reduction in the rate of severe to moderate CP.

A recent meta-analysis suggests that the prenatal administration of antenatal corticosteroids for fetal lung maturity is associated with a reduction in the rate of CP. This meta-analysis included 14 studies, and the antenatal administration of corticosteroids was associated with a significant reduction in the risk of CP, especially in neonates born prior to 28 weeks of gestation (96). Therefore, the current evidence suggests that antenatal treatment with corticosteroids and MgSO_4_ is associated with a significant reduction in the subsequent development of CP in preterm neonates, especially those who were born prior to 32 weeks of gestation. Further study is needed for the better identification of the specific patient population who will benefit from such an intervention in particular.

### Postexposure Treatment of the Affected Neonate

The third option for intervention to prevent CP is the treatment of the affected neonate postexposure. This treatment is more etiology driven than the previous modalities. Indeed, the administration of progesterone for the prevention of recurrent preterm birth is done across the board to all patients with a history of prior spontaneous preterm birth, and all women with threatened preterm delivery receive MgSO_4_, regardless of the etiology of the preterm parturition. This is not the case of postexposure interventions, for example, cooling has become a standard of care, and the hallmark of treatment of neonate who had birth asphyxia (97, 98). Yet, this topic is extensively reviewed elsewhere (99).

Since EOP is both destructive and developmental in origin, therapeutic interventions—both early neuroprotective (that were discussed above) and later restorative—must address this complexity. Restorative therapies are evolving recently in animal models and include, for example, (1) glial-restricted precursor (GRP) cell transplantation in an ischemic neonatal WMI mouse model. Transplantation did not enhance survival, but behavioral and neuropathological outcomes were improved after GRP transplantation (100). (2) Nanomedicine is a new frontier in the development of therapies for the treatment of brain injury resulting in CP. Nanomaterials such as dendrimers provide opportunities for the targeted delivery of multiple drugs that can mitigate several pathways involved in injury and can be delivered specifically to the cells that are responsible for injury (101). (3) Allogeneic umbilical cord blood (UCB) has therapeutic potential for CP. An experimental study in an animal model of CP showed therapeutic effects of intraperitoneally administered UCB cells, with the incorporation of these cells into the brain lesion (102). Concomitant administration of recombinant human erythropoietin may boost the efficacy of UCB, as it has neurotrophic effects (103). The window for restorative therapy depends on the therapy itself as described above, and to this date, there are no restorative therapies that were proven to help CP patients.

This area is still experimental in most and based on animal models and will not be furthermore discussed in this article.

Another emerging topic in the area of postexposure therapy is stem cell therapy. Recent studies researched the use of embryonic stem cells (EST) in the treatment of CP. EST were found to improve the sight of children with CP and cortical visual impairment. Out of 40 children in the study, 39 have shown improvement in sight after this treatment (104). UCB was found to have a small statistically significant intervention on gross motor skills of children corn with CP (105).

## Conclusion

Cerebral palsy is a neurologic motor disorder that can be the result of several underlying mechanisms during intrauterine life or at the newborn period. The risk for CP is gestational age dependent, and it is much more prevalent among preterm neonates, especially those who were extremely premature. Treatments and intervention that prevent preterm birth or assist the premature fetus during parturition, and postnatal intervention, may reduce the incidence of this syndrome in the near future. Moreover, emerging evidence suggests that the understanding of the mechanisms leading to CP will facilitate targeted treatment for the affected child.

## Author Contributions

MS, OM, SM, and OE conceived and wrote the manuscript. NGT and SG wrote the manuscript.

## Conflict of Interest Statement

The authors declare that the research was conducted in the absence of any commercial or financial relationships that could be construed as a potential conflict of interest.
